# Gender differences in white matter pathology and mitochondrial dysfunction in Alzheimer’s disease with cerebrovascular disease

**DOI:** 10.1186/s13041-016-0205-7

**Published:** 2016-03-17

**Authors:** Xavier Gallart-Palau, Benjamin S. T. Lee, Sunil S. Adav, Jingru Qian, Aida Serra, Jung Eun Park, Mitchell K. P. Lai, Christopher P. Chen, Raj N. Kalaria, Siu Kwan Sze

**Affiliations:** Division of Chemical Biology & BioTechnology, School of Biological Sciences, Nanyang Technological University, 60 Nanyang Drive, Singapore, 637551 Singapore; Department of Pharmacology, Yong Loo Lin School of Medicine, National University of Singapore, Singapore, Singapore; Memory, Aging and Cognition Centre, National University Health System, Singapore, Singapore; Institute for Ageing and Health, NIHR Biomedical Research Building, Newcastle University, Campus for Ageing and Vitality, Newcastle upon Tyne, NE4 5PL UK

**Keywords:** Alzheimer’s disease, Cerebrovascular disease, Dementia, Temporal lobe, White matter, Citrullination, Deamidation, Proteomics, iTRAQ

## Abstract

**Background:**

Dementia risk in women is higher than in men, but the molecular neuropathology of this gender difference remains poorly defined. In this study, we used unbiased, discovery-driven quantitative proteomics to assess the molecular basis of gender influences on risk of Alzheimer’s disease with cerebrovascular disease (AD + CVD).

**Results:**

We detected modulation of several redox proteins in the temporal lobe of AD + CVD subjects, and we observed sex-specific alterations in the white matter (WM) and mitochondria proteomes of female patients. Functional proteomic analysis of AD + CVD brain tissues revealed increased citrullination of arginine and deamidation of glutamine residues of myelin basic protein (MBP) in female which impaired degradation of degenerated MBP and resulted in accumulation of non-functional MBP in WM. Female patients also displayed down-regulation of ATP sub-units and cytochromes, suggesting increased severity of mitochondria impairment in women.

**Conclusions:**

Our study demonstrates that gender-linked modulation of white matter and mitochondria proteomes influences neuropathology of the temporal lobe in AD + CVD.

**Electronic supplementary material:**

The online version of this article (doi:10.1186/s13041-016-0205-7) contains supplementary material, which is available to authorized users.

## Background

Dementia prevalence and severity in women are significantly higher than in men after controlling for expected lifespans [1, 2], but the neuropathological basis of this gender bias is currently unknown. Alzheimer’s disease is the most common form of dementia in the elderly, the majority of whom also undergo variable cerebrovascular disease (AD + CVD) [3]. Affected individuals display a complex brain pathology characterized by senile plaques with microinfarcts, as well as a poorly understood degeneration of the small vessels that supply blood to the brain [4–7]. While vascular involvement appears to be closely linked with the extent of white matter (WM) pathology [8–10], it remains poorly defined how small vessel disease influences the clinical course of AD + CVD.

Restriction of blood flow to the brain results in WM tissue damage, leading to neurodegeneration and eventual dementia [11–15]. WM pathology contributes to cognitive impairment and long-term disability in elderly subjects via multiple mechanisms that do not depend on amyloid formation [16, 17]. Indeed, brain WM exhibits a complex composition of different molecules that can be modified to influence brain function, including both lipid molecules and proteins such as 2′,3′-cyclic nucleotide 3′-phosphodiesterase (CNP), myelin proteolipid protein (PLP), and myelin basic protein (MBP) [18]. CNP and PLP, are involved on the maturation of oligodendrocytes [19, 20], while MBP regulates myelination and initiates essential signaling pathways in brain cells [21]. Brain MBP is known to be susceptible to degenerative protein modifications (DPMs) including deamidation and citrullination [22, 23], and myelin degeneration can directly impair cognitive function due to disruption of neuronal circuits [24, 25], but the molecular profile of brain myelin proteins in AD + CVD has yet to be deciphered.

Degeneration of myelin sheaths causes axon disintegration, leading to impaired mitochondrial function and decreased provision of the essential molecules needed to maintain WM integrity [26–28]. Previous studies have also identified that changes in the molecular composition of MBP influence white matter pathology in vascular dementia (VaD) [15, 22, 29], and that excess DPMs promote proteinopathy and neurodegeneration in both AD and VaD [30]. However, it remains unclear to what extent white matter pathology depends on DPM-induced proteinopathy in AD-related human dementias. Intriguingly, a previous study reported gender-specific differences on the accumulation of deamidation in rodent brain proteins [31]. Similarly, prevalence and severity of citrullination-associated diseases in the central nervous system is significantly higher in women than in men [32] what suggests that these two DPMs could also underpin the increased dementia risk observed in human females. We therefore used discovery-driven quantitative proteomics [33] to investigate the molecular basis of gender influences on the pathology of AD + CVD.

## Results

### White matter pathology is influenced by gender in AD + CVD

In order to assess the molecular basis of gender influences on risk of Alzheimer’s disease with cerebrovascular disease (AD + CVD), we first assessed the extent of myelin rarefaction/density loss in post-mortem brain tissues from male and female patients.

**Fig. 1:**
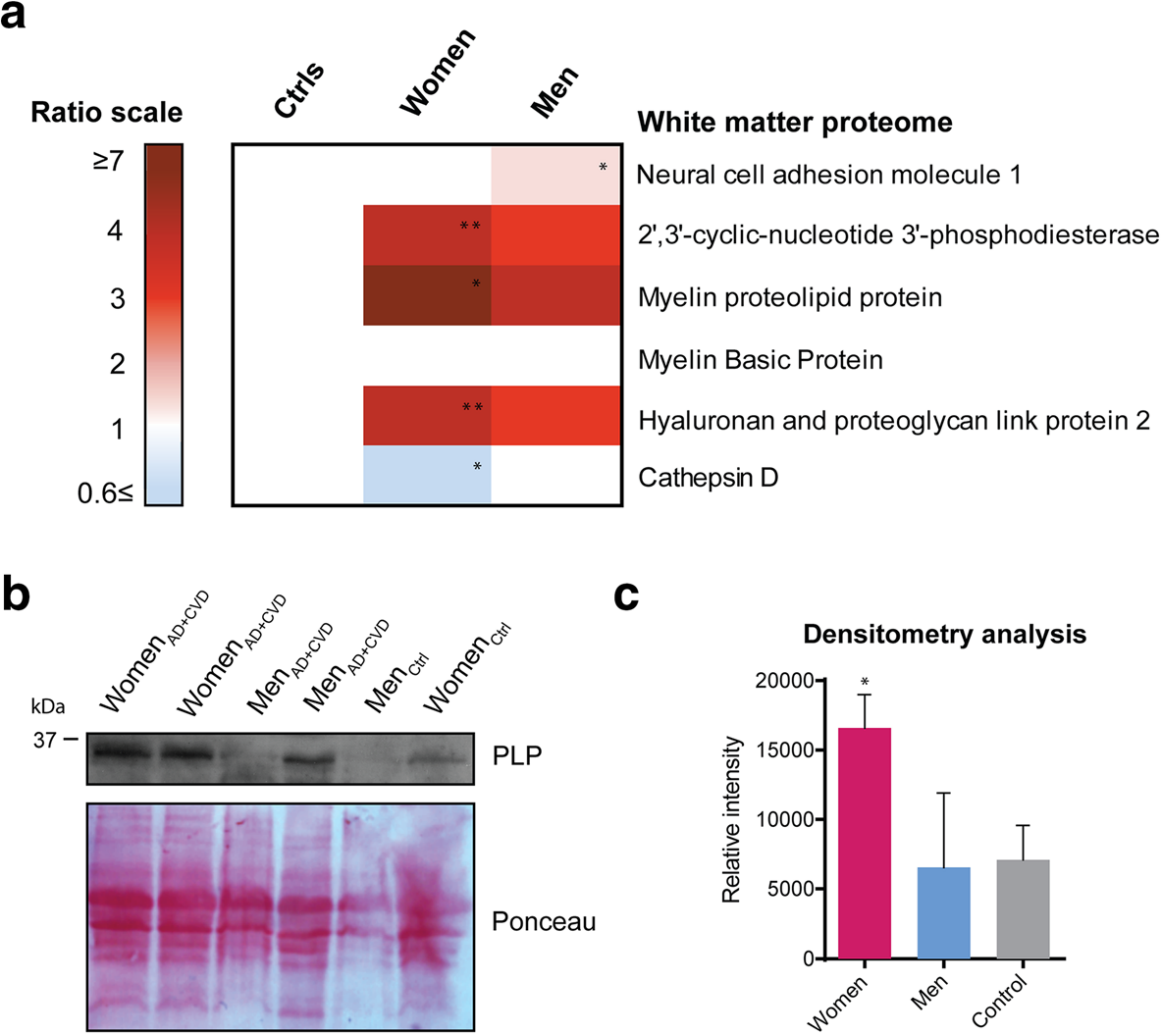
**a** Heatmap displaying protein enrichment/depletion in the white matter proteome of male and female AD + CVD subjects compared to age-matched controls. Log ratios in AD + CVD subjects were normalized to controls (controls ratio = 1). Red values represent protein enrichment, blue values represent protein depletion and white values indicate no change in the ratio scale. Protein names were listed on the right side of each heat map line. Significance level of modified proteins in each group were indicated at the upper right corner of each box (* *p* < 0.05 and gender difference ≥ 0.2; ** *p* < 0.001 and gender difference ≥ 0.9). Tabular data of values shown in this heat map can be found in Additional file 1: Table S1. **b** Validation of the PLP enrichment in the temporal lobe of AD + CVD women was confirmed by Western blot in individual subjects from an independent cohort. **c** Densitometry analysis of Western blot signal normalized by Ponceau intensities. Significance level in graph of PLP signal in women relative to controls (* p < 0.05)﻿

While there were no gender-specific differences noted during post-mortem evaluation of brain myelin density in AD + CVD (Table 1), analysis of the temporal lobe proteome of dementia patients revealed significant up-regulation of myelin proteins CNP, PLP and hyaluronan proteoglycan 2 (HPLN2), which were further increased only in female patients (Fig. 1a, Additional file 1: Table S1). PLP enrichment in the temporal lobe of female AD + CVD was also validated in another independent cohort by western blot (Fig. 1b and c).

**Table 1:** Demographic and clinical data of dementia subjects and age-matched control subjects. 1. Post-mortem delay

Age-matched control subjects							
Gender	Age	PM^a^ delay	Cognitive assessment	Cog. exam.	Braak	CERAD	Neuropathology
M	72	17	Normal control	Retrospective Interview	1	Sparse	Mild AD-like pathology
M	75	20	Normal control	Retrospective Interview	0	None	Scattered microinfarcts (in right hemisphere: hippocampal CA2/3, putamen, caudate, external medullary lamina) and SVD: Foci of arteriosclerosis mainly in white matter
F	68	75	Normal control	Retrospective Interview	1	Sparse	Mild cerebral amyloid angiopathy. A right frontal microinfarct and small lacunar infarct in the left putamen. Infarcts in both cortex and basal ganglia
F	78	23	Normal control	Retrospective Interview	2	Sparse	Mild AD-like pathology
F	82	26	Normal control	Retrospective Interview	0	N.A.	No detected AD or CVD pathology
F	87	14	Normal control	Retrospective Interview	N.A.	N.A.	Low mean density of neocortical tangles = 2.5 per mm2.
F	99	19	Normal control	Retrospective Interview	N.A.	None	No detected AD or CVD pathology
F	86	40	Normal control	Retrospective Interview	1	N.A.	No detected AD or CVD pathology
F	75	31	Normal control	Retrospective Interview	N.A.	N.A.	No detected AD or CVD pathology
F	81	51	Normal control	Retrospective Interview	N.A.	N.A.	No detected AD or CVD pathology
Mean	80.3 ± 8.9	31.6 ± 19					
AD + CVD subjects							
Gender	Age	PM^a^ delay	Cognitive assessment	Cog. exam.	Braak	CERAD	Neuropathology
M	83	71	1 y. cog imp.	M.M.S.E = 18	4	Moderate	Mixed pathology - AD and SVD (predominantly microinfarcts in frontal lobe & perivascular lacunae in basal ganglia). Cavernous hemangioma in right ventrolateral posterior centrum semiovale. Loss of myelin in brain capsules.
M	72	29	7 y. cog imp.	M.M.S.E = 20	4	Frequent	Mixed AD and CVD. Microinfarcts in putamen. Loss of myelin in internal capsule
M	84	42	8 y. cog imp.	M.M.S.E = 13	5	N.A.	Mixed pathology AD with vascular dementia and severe cerebral amyloid angiopathy.
M	82	38	1 y. dementia	M.M.S.E = 16	6	N.A.	Mixed pathology AD with old infarcts in the temporal cortex right hemisphere. CVD affecting parietal and occipital lobes. Microinfarcts in caudate and putamen. Focal loss of myelin in brain capsules.
M	64	24	1 y. dementia	M.M.S.E = 20	N.A.	N.A.	Mixed pathology AD + CVD affecting middle temporal gyrus, parietal and occipital lobes. Myelin affectation in brain capsules.
Mean	77 ± 8.7	41 ± 18					
F	93	18	2 y. dementia	S.I.B = 75/100	5	Frequent	Mixed pathology AD + CVD affecting parietal and temporal lobes. Myelin affectation in brain capsules.
F	89	72	4 y. cog. imp.	M.M.S.E = 16	4	N.A.	Mixed pathology AD + CVD affecting temporal lobes and brainstem. Severe myelin affectation in medial and internal temporal gyrus.
F	89	32	3 y. cog. imp.	M.T.S = 5/37	5	N.A.	Mixed pathology AD + CVD. Severe changes in hippocampus. Neurofibrillary tangles in raphe nucleus.
F	92	12	5 y. dementia	M.M.S.E = 20	5	N.A.	Mixed pathology AD + CVD affecting middle temporal gyrus and parietal/occipital lobes. Severe myelin affectation in temporal cortex.
F	86	99	2 y. dementia	M.M.S.E = 20	6	N.A.	Mixed pathology AD + CVD. Moderate temporoparietal infarcts.
Mean	89.8 ± 2.8	46.6 ± 37.5					

^a^Post-mortem delay

Intriguingly, we observed down-regulation of the myelin-associated protein Cathepsin D in women with dementia while the rest of myelin proteins identified in that group were significantly upregulated (Fig. 1a). Furthermore, the protein neural cell adhesion molecule (NCAM1) involved on functional response to white matter injury was only up-regulated in male patients (Fig. 1a). These data indicated that unbiased quantitative profiling of the human brain proteome can detect gender-specific differences in white matter pathology from patients with AD + CVD that were not apparent from conventional post-mortem evaluation.

The brain protein myelin associated glycoprotein (MAG) is highly susceptible to ischemia-induced degradation and its levels do not vary in the mammal’s brain by the effect of gender [34]. Similarly, PLP positively correlates with severity of white matter pathology in small vessel disease [35], hence MAG/PLP ratio can be used as a proxy measure of disease severity in affected patients [35, 36]. Using this approach in our dementia samples, we observed that MAG/PLP ratio was significantly lower in females (0.2) than in males (0.5), indicating greater severity of WM pathology in women with AD + CVD. Since both genders exhibited comparable expression levels of MAG protein, which is readily degraded under ischemic conditions [35], these data suggested that both men and women may undergo a similar extent of ischemic injury in AD + CVD, but that subsequent effects on the myelin proteome and PLP expression differ between genders.

### Sex-influenced modification of myelin basic protein in AD + CVD

**Fig. 2:**
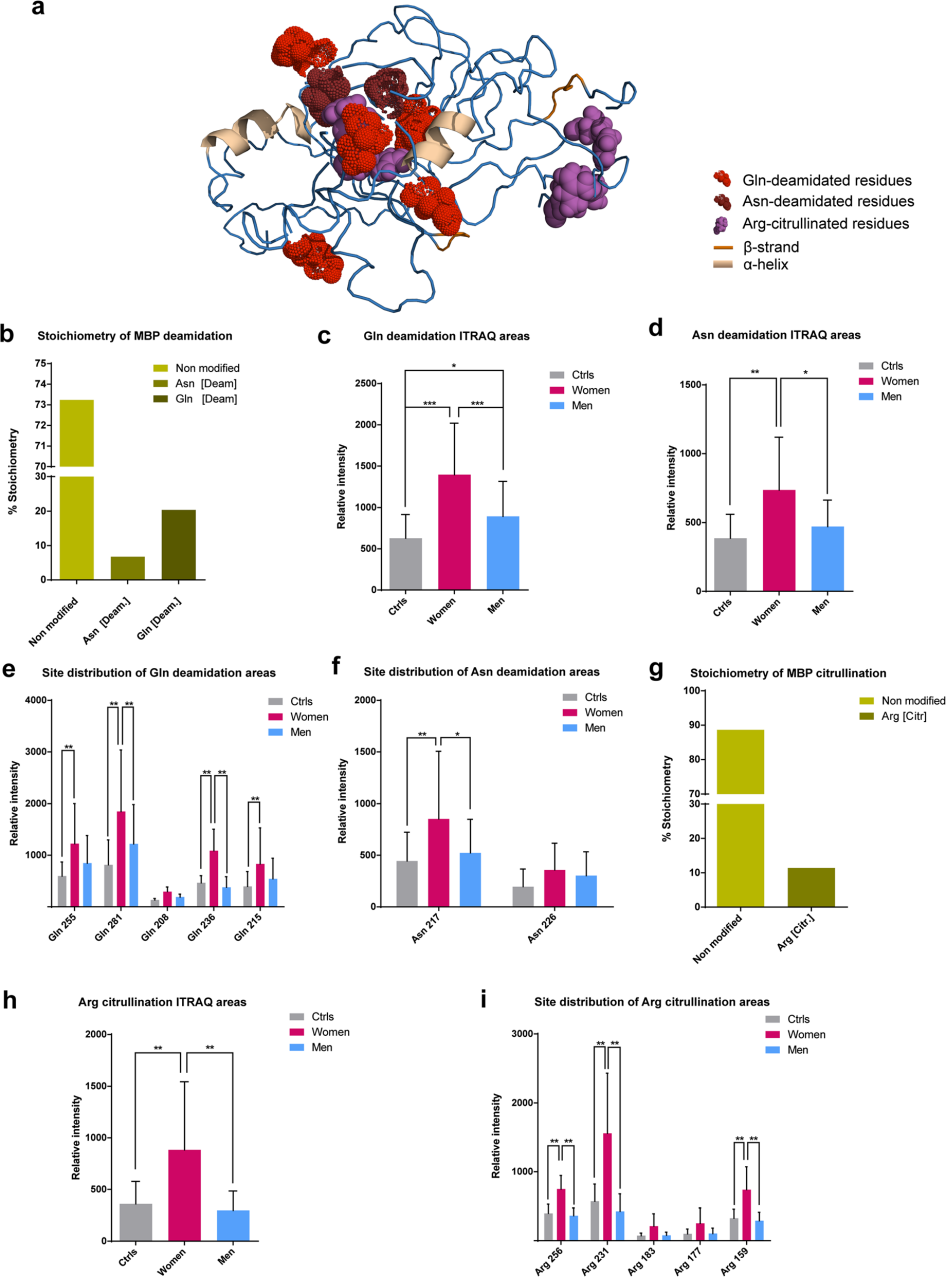
Degenerative modifications in MBP. **a** 3D structural modelling of MBP sequence in AD + CVD, showing modified sites including Arg citrullination and Gln/Asn deamidation. **b** Stoichiometry of MBP deamidation considering the percentage of all confidentially identified peptides in all groups (AD + CVD subjects and controls). **c** Relative level of Gln deamidation considering all the identified sites from the obtained iTRAQ peak intentities in age-matched controls, AD + CVD women and AD + CVD men. **d** Relative levels of Asn deamidation considering all the identified sites from the obtained iTRAQ peak intentisites in age-matched controls, AD + CVD women and AD + CVD men. **e** Site distribution of Gln deamidation peak intensities in age-matched controls, AD + CVD women and AD + CVD men. **f** Site distribution of Asn deamidation peak intensities in age-matched controls, AD + CVD women and AD + CVD men. **g** Stoichiometry of MBP citrullination considering the percentage of all confidentially identified peptides in all groups (AD + CVD subjects and controls). **h** Relative level of Arg citrullination across all sites based on the obtained iTRAQ areas in age-matched controls, women with dementia, and men with dementia. **i** Site distribution of Arg citrullination iTRAQ area intentities in MBP from age-matched controls, women with dementia, and men with dementia. Significance level in graphs (* *p* < 0.05; ** *p* < 0.001)

Degenerative protein modifications (DPMs) including deamidation and citrullination are thought to underpin loss of protein function in the brains of dementia patients [37–40], so we next assessed whether protein modification profiles differed between male and female AD + CVD. To do this, we performed functional proteomic analysis by studying the quantitation iTRAQ reporter area of all modified peptides for each identified peptide of the BA21 brain proteome. Using our unbiased discovery approach, we identified that myelin basic protein (MBP) exhibited the most extensive occurrence of DPMs in whole proteome of AD + CVD subjects as mapped in Fig. 2a. Stoichiometry of deamidation at glutamine (Gln) and asparagine (Asn) residues covered ~30 % of all identified MBP constituent peptides (Fig. 2b). When compared with controls, AD + CVD patients exhibited increased ratio of deamidation in brain MBP, and this was more extensive in women than in men (Fig. 2c and d), particularly at residues Gln 281, 255, 236 and 215 (Fig. 2e), suggesting increased incidence of DPMs in women with dementia. Consistent with this concept, we also observed significantly higher deamidation of Asn 217 in brain MBP only in female AD + CVD patients (Fig. 2f). Further stoichiometric analysis revealed that citrullination of arginine (Arg) residues affected ~12 % percent of MBP constituent peptides in AD + CVD (Fig. 2g), and we detected hyper-citrullinated MBP only in female patients (Fig. 2h). Citrullination of Arg residue 256 was significantly increased in women with dementia (Fig. 2i), which also displayed extensive citrullination of Arg residues 159 and 231, whereas DPM of these residues did not differ between male dementia patients and controls (Fig. 2i). These data suggested that women with AD + CVD exhibit significantly higher brain protein DPMs than male patients or controls.

### Sex-influenced degradation of myelin basic protein in AD + CVD

**Fig. 3:**
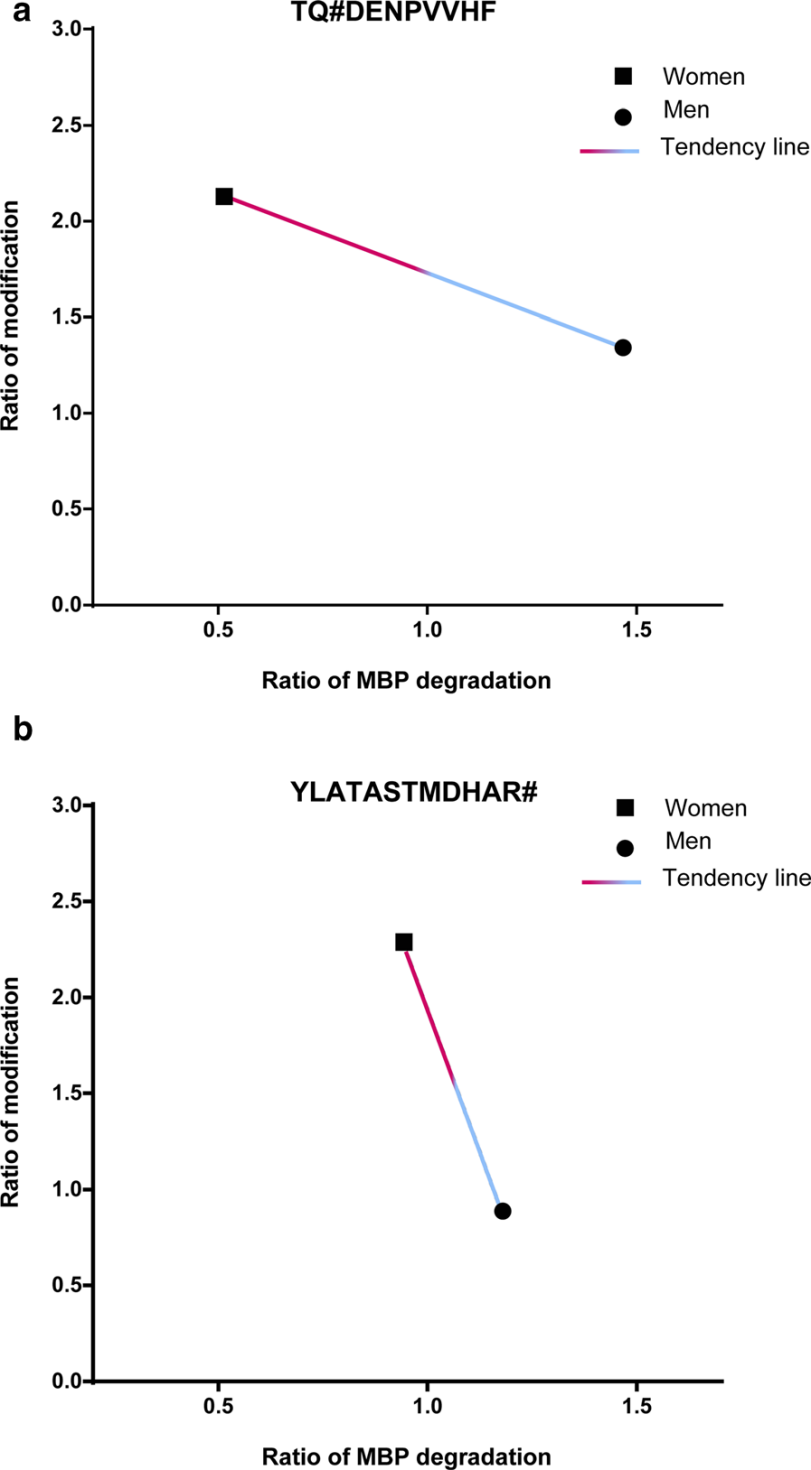
Degradation of MBP in the temporal lobe of AD + CVD subjects. Degradation ratio of MBP was calculated based on the peptide intensity areas in each group showing exact match with previous reported MBP degradation byproducts by the effect of cathepsin D (see detailed explanation of that method in the material and methods section). All ratios were normalized to age-matched controls (ratio 1 = normal degradation). **a** Degradation ratio of the MBP byproduct TQ#DENPVVHF in women and men with dementia. As shown, higher ratio of Gln deamidation (>2.0) in the MBP byproduct TQ#DENPVVHF was associated with lower degradation ratio (<0.5) of the protein in AD + CVD women. **b** Degradation ratio of the MBP byproduct YLATASTMDHAR# in women and men with dementia. MBP degradation ratio of this byproduct in AD + CVD women (≈1) was not affected by the increased level of citrullination (ratio ≈ 2.5)

Since WM pathology in AD and VaD is associated with accumulation of degenerated MBP (dMBP) [15], we next assessed whether the sex-specific DPMs detected here could influence the degradation profile of brain MBP. In dMBP, the protein sequence QDENPVV (residues 82–88) is susceptible to degradation by cathepsin D due to the 3D structure of dMBP and generates the byproduct peptide TQDENPVVHF (residues 81–90) [41–43]. We therefore assessed whether women and men with AD + CVD exhibited differential deamidation of Gln residue 82 in the exposed region of dMBP. Using this approach, we observed that women with dementia displayed increased stoichiometry of deamidation in Gln 82, which was associated with impaired degradation of this protein region (Fig. 3a) and accumulation of dMBP. These data suggested that dMBP due to Gln deamidation may be more resistant to proteolytic cleavage similar to other proteins reported previously [30, 44] and to removal from brain tissues of female AD + CVD patients. In contrast, AD + CVD patients did not exhibit any change on the degradation ratio of the cathepsin D byproduct YLATASTMDHAR#, despite that increased level of peptide citrullination was detected for this byproduct in women with dementia (Fig. 3b). These data indicated that sex-specific degradation of brain proteins in AD + CVD varies across the proteome in BA21, with MBP being the most heavily modified protein in the brain of female with dementia.

### Female AD + CVD is associated with specific modifications of the temporal lobe and mitochondria proteomes

**Fig. 4:**
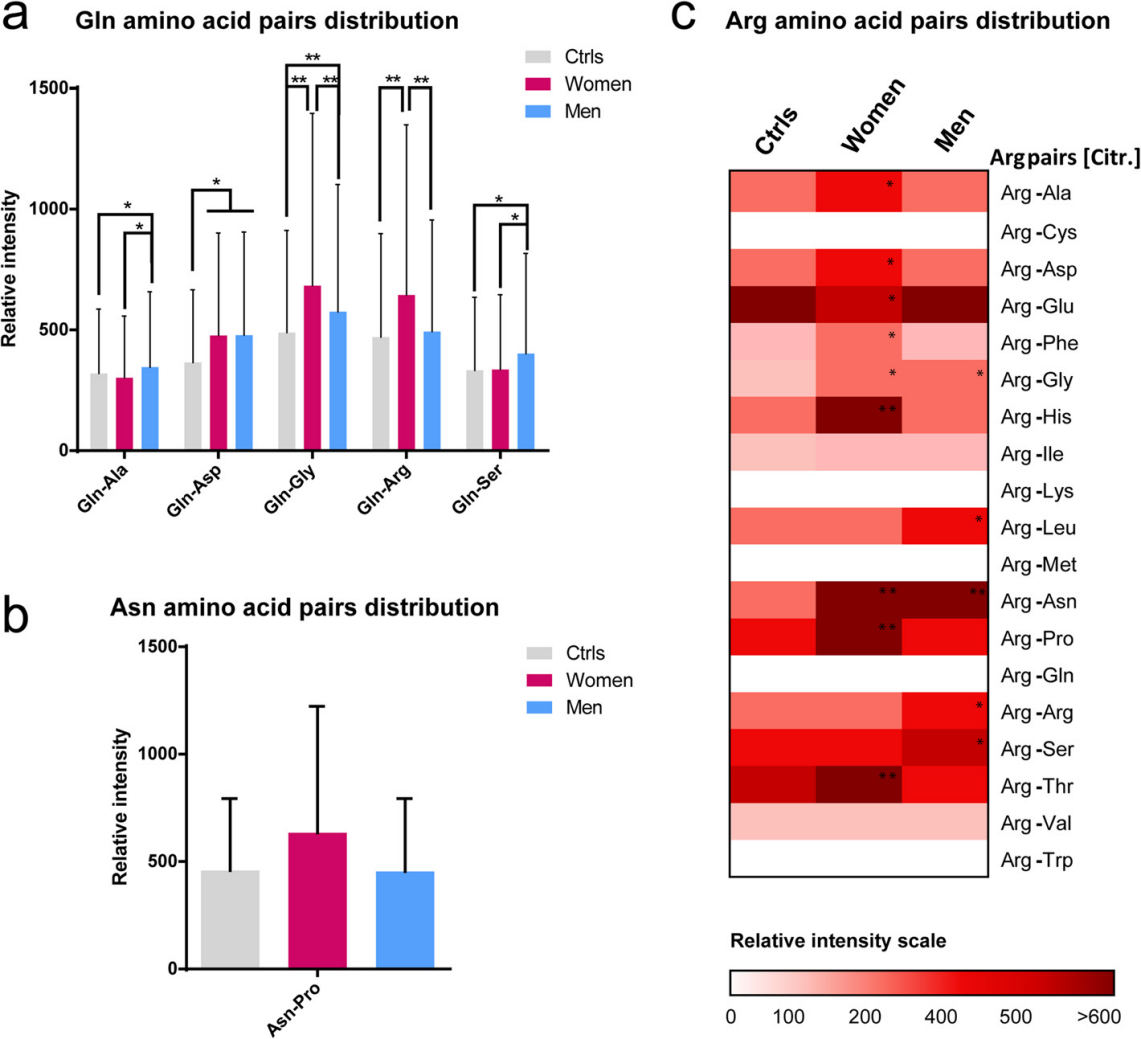
Characterization of modified amino acid pairs in whole temporal lobe proteome of AD + CVD subjects and controls. **a** Relative intensity areas of deamidated Gln amino acid pairs in the temporal lobe proteome of age-matched controls, women with dementia, and men with dementia. **b** Relative intensity areas of deamidated Asn amino acid pairs in the temporal lobe proteome of age-matched controls, women with dementia, and men with dementia. **c** Heat map displaying relative intensity areas of Arg citrullination amino acid pairs in whole temporal lobe proteome of age-matched controls, women with dementia, and men with dementia. In the heat map ratio scale red values indicate up-regulation of the referred amino acid pair and white values indicate no identification. The following amino acid pairs were not susceptible to Arg citrullination in the temporal lobe of human subjects; Arg-Cys, Arg-Lys, Arg-Met, Arg-Gln and Arg-Trp. Significance level between AD + CVD and controls (* *p* < 0.05; ** *p* < 0.001)

Women with cognitive disorders have been reported to display impaired glutamine-glutamate metabolism in the brain [45, 46], and may exhibit mitochondria dysfunction associated with defective hormone signaling [47], so we next sought to identify sex-specific modulation of the temporal lobe and mitochondria proteomes in AD + CVD. Whole temporal lobe proteome displayed increased levels of Gln deamidation in AD + CVD subjects relative to age-matched controls (3.90 % female dementia, 3.87 % male dementia, 3.52 % age-matched controls; chi square 27.698 with one degree of freedom, *p*-value <0.001) and in female patients we detected increased levels of the enzyme carbonyl reductase 1 (NADPH1) compared with either male patients or controls (iTRAQ ratio female/controls 2.09 ± 0.13; *p*-value <0.001; gender ratio female/male 2.36). Since NADPH1 can promote deamidation of Gln residues [48], and we detected a reduction in glutamine synthetase (GS) levels in women with dementia (iTRAQ ratio female/controls 0.5 ± 0.09; *p*-value 0.002; gender ratio female/male 0.45), these data could explain the impaired glutamine-glutamate metabolism identified in the temporal lobe of female dementia patients. Indeed, we also found numerous gender-specific deamidations of various Gln amino acid pairs and a single asparagine amino acid pair in the temporal lobe of AD + CVD subjects (Fig. 4a and b). We also observed increased citrullination of Arg residues in female AD + CVD patients compared with male patients, but only at a limited number of amino acid pairs (Fig. 4c).

**Fig. 5:**
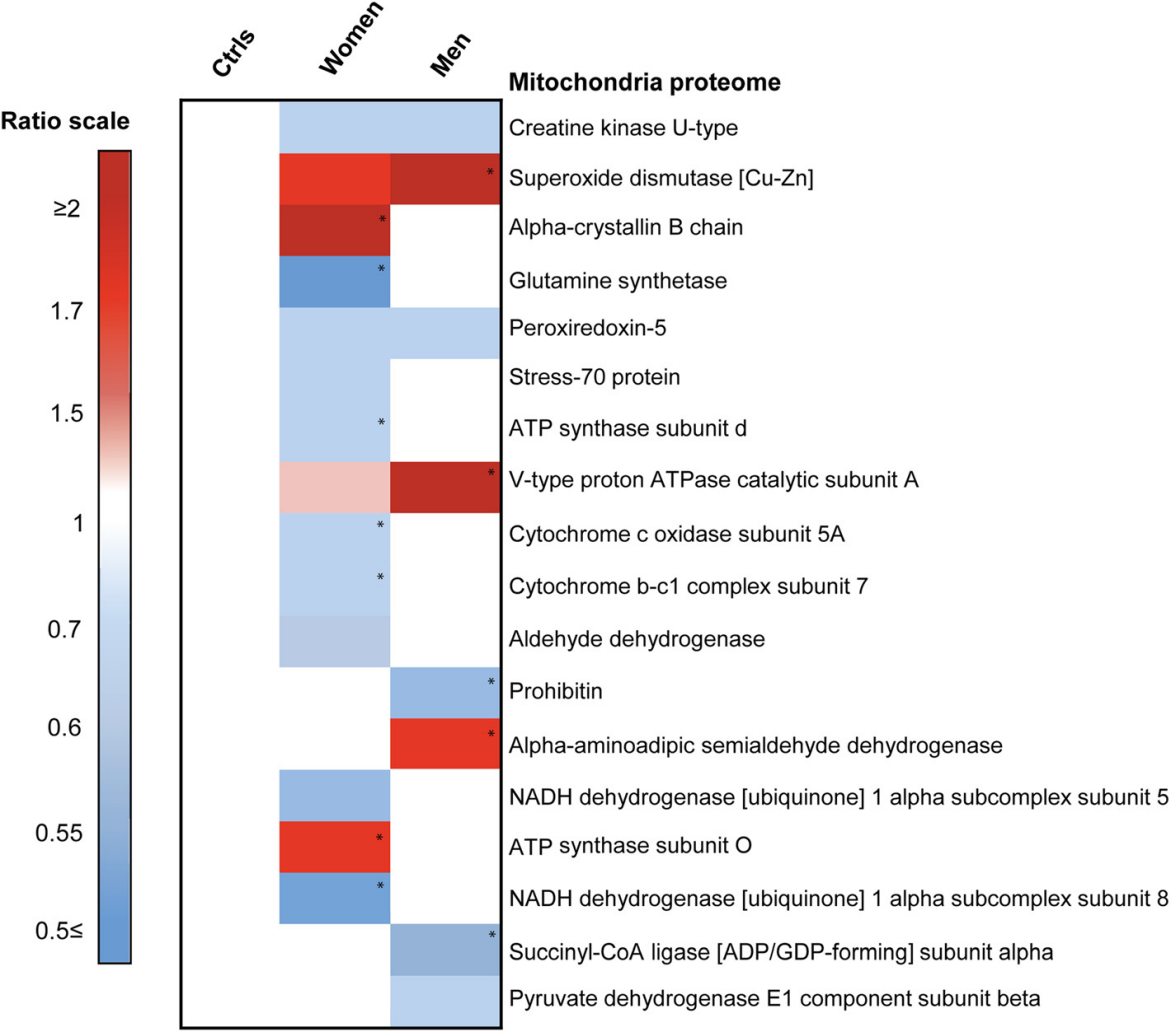
Heatmap showing ratios of protein enrichment/depletion in the mitochondrial proteome of male and female AD + CVD subjects compared to age-matched controls. Log ratios in AD + CVD subjcets were normalized to controls (controls ratio = 1). Red values represent protein enrichment, blue values represent protein depletion and white values indicate no change in the ratio scale. Protein names were listed on the right side of each heat map line. All proteins reported here are significantly modified versus controls (*p* < 0.05). Significance level between AD + CVD groups (* *p* < 0.05 and gender difference ≥ 0.2). Tabular data of values shown in this heat map can be found in Additional file 2: Table S2

Dementia pathology is strongly associated with mitochondrial production of reactive oxidative species (ROS), and in our AD + CVD samples we detected up-regulation of the dismutase protein superoxide dismutase-1 as well as down-regulation of aldehyde dehydrogenase and mitochondrial creatine kinase-U-type relative to controls (Fig. 5, Additional file 2: Table S2). While evidence of mitochondria dysfunction was apparent in both men and women with dementia, female patients also exhibited dysregulation of the D and O subunits of ATP synthase, together with down-regulation of cytochromes, NADH ubiquinone alpha subunits (5 and 8) and chaperone stress-70 (Fig. 5, Additional file 2: Table S2), indicating that perturbation of the mitochondria proteome in AD + CVD is more pronounced in women than in men. Together, these data confirm a positive association between WM pathology and mitochondria dysfunction in the temporal lobe of AD + CVD subjects, and suggest that dysregulation of mitochondrial ATP synthesis; redox activity and cytochrome function represent gender-specific neurodegenerative processes in AD + CVD.

## Discussion

In the current study, we used discovery-driven quantitative proteomics to uncover gender influences on myelin neuropathology and dysfunctional mitochondria proteomes in the temporal lobe of patients with AD + CVD. The data from this study provide novel insight into the molecular basis of the increased dementia risk and disease severity observed in women that develop AD + CVD.

Despite that post-mortem evaluation of myelin density failed to detect gender differences on white matter pathology in patients with AD + CVD, the data from quantitative profiling of the brain proteome clearly revealed the gender-specific molecular pathology of the affected WM. While we detected similar levels of MAG protein in men and women with dementia, suggesting a comparable extent of ischemic injury in both genders based on MAG/PLP ratio [35, 36], the severity of WM pathology observed in women was greater than that observed in men. Accumulation of dMBP in the temporal lobe is a key indicator of WM pathology in dementia [15, 49]. In disease settings, hyper-citrullination of MBP is thought to increase protein degradation by cathepsin D and other enzymes, leading to axonal dysfunction and progressive loss of neuronal function [43, 50, 51]. In-line with predictions, our functional proteomics study confirmed that MBP was hyper-citrullinated in women with dementia, but we also observed an unexpected impairment of dMBP degradation, which was associated with reduced cathepsin D expression and increased MBP deamidation at Gln residues (particularly in the degenerative epitope QDENPVV) [15, 41, 42]. Deamidation of Gln residues favors the proteolysis of dysfunctional proteins via the ubiquitin proteasome system [52], but Gln deamidation of brain proteins including MBP is also strongly associated with proteinopathy [51, 53, 54] which may resist the degradation of dMBP by the ubiquitin proteasome system. This impaired clearance of dMBP leads to accumulation of dysfunctional protein in the female brain. Our data now indicate that gender influences on the dementia-associated deamidation of MBP may alter protein degradation in the temporal lobe of AD + CVD patients.

We also observed marked up-regulation of several different myelin proteins in the temporal lobe of AD + CVD subjects, suggestive of an ongoing yet dysfunctional remyelination process [55]. Our data are therefore consistent with a previous report that WM pathology is associated with accumulation of HPLN2, which inhibits axonal remyelination in the brain [55]. Abnormal remyelination may also account for the counterintuitive increase in several myelin proteins including CNP and PLP in the temporal lobe of AD + CVD subjects. Impaired remyelination has previously been characterized to be associated with WM lesions [56], and may also be a feature of patients with AD + CVD, in whom the putative remyelination defect was closely associated with Gln deamidation of MBP.

In a recent study of mice that lack the enzyme L-isoaspartyl methyltransferase (PIMT) which repairs damaged proteins, female animals were reported to exhibit greater accumulation of IsoAsp-type DPMs in the brain [31] . PIMT knock-out mice have also been shown to exhibit increased deamidation and imbalance of the glutamate-glutamine cycle in the brain [57]. These data are consistent with the current report, which suggests that gender-associated increases on deamidation of specific brain proteins may contribute to the increased severity of AD + CVD observed in women. While gender influences on enzymatic citrullination of Arg residues were less marked than effects on Gln deamidation by NADPH1, both processes can liberate ammonia byproducts thought to contribute to WM damage in human dementia [48, 58]. Accordingly, we also detected increased NADPH1 levels together with down-regulation of GS in the temporal lobe of women with AD + CVD. GS are astrocytic enzymes able to efficiently capture free ammonia during the Gln synthesis in the brain [59, 60]. Down-regulation of GS in the temporal lobe of women with dementia may indicate abnormal production of Gln in the brain, in-turn leading to increased expression of NADPH1 enzymes that enhance production of glutamate from Gln residues. Further research will now be required to assess this possibility.

Whether mitochondria dysfunction precedes changes in the WM proteome or *vice versa* remains unclear. Other colleagues have reported that an increase in ammonia byproducts during neurodegeneration can exacerbate glutamate toxicity and impair mitochondria function [61]. Here we observed that AD + CVD patients displayed down-regulation of mitochondrial kinase-U-type protein, which is known to be dysregulated in ischemia-induced mitochondrial impairment [62]. We further observed that cytochromes and ATP subunits were significantly altered only in women with AD + CVD, consistent with increased severity of mitochondria dysfunction in this group. According to our findings, perturbation of the mitochondrial proteome appears to be proportional to the severity of WM pathology in human AD + CVD. This result suggests that mitochondria dysfunction in AD-related disorders may be a product of early alterations in the WM proteome by vascular dysfunction, hence as recently suggested the vascular component of disease is likely to exert a major influence on the clinical course of human dementias.

## Conclusions

While several epidemiological and clinical studies have showed that women exhibit higher risk of dementia than men, the molecular neuropathology of this gender difference remains elusive. In the current study, we used unbiased quantitative proteomics to assess the molecular basis of gender influences on risk of AD + CVD. For the first time, we report sex-specific molecular differences in white matter pathology and mitochondrial proteomes in the temporal lobe of AD + CVD patients. In particular, we observed that hyper-citrullination and hyper-deamidation of MBP were prevalent in female dementia patients. Specifically, deamidation of the glutamine residue 82 in the MBP degenerative epitope was associated with impaired degradation and accumulation of degenerated protein in the temporal lobe of women with dementia. This study uncovers the gender influences on the neuropathology of AD + CVD, and may pave the way for future clinical interventions that can reduce dementia risk in both male and female patients.

## Methods

### Brain tissues

Autopsied brain specimens were carefully evaluated for the presence of senile plaques and CVD at the Newcastle Brain Tissue Resource (NBTR, UK). The temporal cortex region BA21 was used in all experiments (both control and dementia samples). All dementia brain samples met histological criteria for AD + CVD, whereas control subjects lacked features of either dementia or AD + CVD. Brain samples in each experimental group were closely matched for key variables including post-mortem delay, cognitive assessment data, age at death, and histological evaluation (Table 1) [63]. Finally, BA21 tissues for western blot validation of the PLP level were generously provided by the Harvard brain tissue resource center (HBTRC). Informed consent was obtained from all participants or their legal representatives. All experimental procedures were approved by the ethical boards at Nanyang Technological University (NTU, Singapore) and NBTR, and were performed in accordance with NTU guidelines.

### Reagents

All reagents were purchased from Sigma-Aldrich (St. Louis, MO, USA) unless specified otherwise.

### Tissue processing and protein extraction

In order to minimize potential confounding factors and to efficiently limit the excessive cost of iTRAQ experiments, we adopted a pooling strategy for our proteomic analyses [64–66]. Approximately 10 milligrams of brain tissue from each subject was homogenized in 1 % SDS buffer using the tissue homogenizer bullet blender (Next Advance, NY, USA) and then pooled into one of the following three groups: age-matched controls (10 subjects), male dementia subjects (5 subjects), and female dementia subjects (5 subjects). Experiments were performed in triplicate, and only those proteins that were confidently identified in all three experiments are reported here (see detailed description of statistical analyses below).

Proteins were acetone-precipitated and quantified by bicinchoninic acid assay. Two-hundred micrograms of protein were resolved by SDS-PAGE and visualized using Coomassie Blue staining. Protein bands were cut and destained in 75 % acetonitrile containing 25 mM triethylammonium bicarbonate (TEAB). Gel cubes were reduced with Tris 2-carboxyethyl phosphine hydrochloride (5 mM), alkylated with methyl methanethiosulfonate (10 mM), and then dehydrated using acetonitrile. Proteins were digested overnight at 37 °C in sequencing-grade modified trypsin (Promega, Madison, WI, USA). Peptides were extracted using 50 % acetonitrile in 5 % acetic acid solution under ultrasound sonication, then dried and concentrated using a vacuum concentrator (Eppendorf AG, Hamburg, Germany).

### iTRAQ labelling and shotgun mass spectrometry

Labeling of dried peptides was performed as previously reported [38, 39, 67]. Briefly, 4-plex iTRAQ reagent Multiplex kits (Applied Biosystems, Foster City, CA) were used according to the manufacturer’s protocol. Tags were distributed as follows; 114 = controls; 116 = women; 117 = men. The iTRAQ-labeled peptides were desalted using Sep-Pak C18 cartridges (Waters, UK) and fractionated by high-performance liquid chromatography (HPLC) (Shimadzu, Kyoto, Japan) on a PolyWAX LP column (4.6 × 200 mm, 5 μm, 300 Å) (PolyLC, Columbia, MD, USA). Buffer A (10 mM ammonium acetate, 85 % acetonitrile, 0.1 % acetic acid) and buffer B (30 % acetonitrile, 0.1 % formic acid) were used to establish a 60 min HPLC-gradient at 1 ml/min flow rate. Chromatograms were recorded at 280 nm. A total of 60 fractions were collected and subsequently combined into 26 fractions according to peak intensities.

LC-MS/MS analysis of the brain peptides was performed using a QSTAR Elite mass spectrometer (Applied Biosystems/MDS Sciex, Foster City, CA, USA) coupled with online nanoflow multidimensional liquid chromatography system (MDLC). A custom-made nanobore C18 column with a picofrit nanospray tip (75 μm ID × 15 cm, 5 μm particles) was used to separate the iTRAQ-labeled peptides. The QSTAR Elite was set to positive ion mode using Analyst QS 2.0 software for data acquisition (Applied Biosystems, Foster City, CA, USA). The precursors with a mass range of 300–1600 m/z and calculated charge from +2 to +5 were selected for fragmentation. Peptides above a 5-count threshold were selected for MS/MS and each selected target ion was dynamically excluded for 20 s with a mass tolerance of 0.1 Da. Smart information-dependent acquisition was activated with automatic collision energy and automatic MS/MS accumulation. The fragment intensity multiplier was set to 20, and the maximum accumulation time was 2 s.

### LC-MS/MS data search

MS/MS data searching was performed using the concatenated target-decoy Uniprot database in ProteinPilot software 3.0 (revision number 114732; Applied Biosystems, Foster City, CA, USA) with Paragon (3.0.0.0, 113442) and Pro Group algorithms implemented. Digestion enzyme was set as semi-trypsin. User-defined parameters and false discovery rate (FDR) for assignation of peptides and proteins in the software were set as previously specified [38]. Briefly FDR <1 % (FDR = 2.0 × [decoy hits/total hits] × 100 %) and unused Protein Score value ≥2 were used as qualification criteria (corresponding to a confidence limit of 99 %). These criteria enabled the identification of over 2400 total proteins.

### Regulation of protein levels and statistical inference

The Pro Group algorithm in Protein Pilot was used to automatically calculate the iTRAQ reporter ratio for each protein based on error factor and *p*-value; Error factor = 10 ^95% confidence error^ where 95 % confidence error = *S*_MW_ × (Student’s *t* factor for *n* – 1° of freedom). *S*_MW_ refers to weighted standard deviation of the weighted average of log ratios, and *n* refers to number of peptides contributing to protein relative quantification. *P-values* were determined by calculating Student’s *t* factor where *t* = (weighted average of log ratios − log bias) divided by weighted standard deviation; which allowed determination of *p*-value with *n* − 1° of freedom. The statistical criteria used by Paragon and Pro Group algorithms in Protein Pilot have been described in detail elsewhere [68, 69]*.* Regulation cut-offs to identify protein differential expression between dementia groups and controls were established based on the calculated percentage coefficient of variation (% CV) of the protein ratios. A total 95 % of the proteins identified in our study displayed a ratio % CV <50 % (Additional file 3: Figure S1A). Accordingly, we set the ratio threshold at >1.5 for protein up-regulation and at <0.67 (1/1.5) for protein down-regulation. No significant differences were observed for % CV of ratios between the male and female dementia groups, indicating successful control of outliers in each respective pool. Furthermore, no significant differences were observed in postmortem delay mean confidence intervals between experimental groups (Additional file 3: Figure S1B). In order to increase confidence in detecting changes in protein expression level, we then combined these data with a G-test calculation [70] for each protein using only those contributor peptides identified with ≥ 99 % of confidence. G-values were calculated as follows:

$$ G=2\times \left(Ctr{l}_A\times ln\left[Ctr{l}_A\div \left(\left(Ctr{l}_A+De{m}_A\right)\div 2\right)\right]+De{m}_A\times ln\left[De{m}_A\div \left(\left(Ctr{l}_A+De{m}_A\right)\div 2\right)\right]\right) $$

In this equation, Ctrl_A_ refers to iTRAQ reporter area in the control group, Dem_A_ refers to iTRAQ reporter area of the respective dementia group, and ‘ln’ refers to the natural logarithm. G-values fit well to the *x*^2^ distribution with one degree of freedom [66], and accordingly, we were able to calculate the corresponding *p*-value for each G-value obtained. Finally, the calculated *p*-values were corrected for multiple comparisons using the Benjamini-Hochberg FDR correction at 0.05α [71] to obtain a corrected *p*-value of 0.014. Following this approach, we considered only those proteins confidently identified in all three experiments (i.e. those containing at least one unique peptide identified with >99 % confidence in each group, and displaying a G-test *p*-value lower than 0.014). A total of 321 proteins satisfied these stringent criteria (Additional file 4) and (Additional file 3: Figure S1C). We then assessed the influence of gender on expression of the 321 proteins identified by analyzing the %CV of the iTRAQ ratios in both the male and female dementia groups. The cut-off for protein differential expression between men and women with dementia was set at ≥0.2 (based on %CV <20 % ([116:114] – [117:114]) (Additional file 3: Figure S1D). Combining all the statistical approaches described above, we observed that a total of 59 proteins were significantly modulated in the dementia groups compared with controls (Additional file 5), including 38 proteins that were differentially expressed between male and female dementia patients (Additional file 6).

### Quantification of peptide modifications and statistical inference

To confidently quantify the level of modified peptides in our profiled AD + CVD brain tissues we assessed influence of age and disease on the increase of deamidation and citrullination in temporal lobe proteins performing spectral counting in peaks [72]. This analysis confirmed the exacerbate increase of DPTMs in temporal lobe proteins as a disease-associated process (Additional file 3: Figure S1E and S1F). Analysis of modified peptides (deamidation and citrullination) was performed using unique peptide iTRAQ reporter peak areas. Only those peptides identified with ≥ 99 % confidence were included in this analysis. Mean peptide reporter peak areas in each of the three groups (age-matched controls, men with dementia, women with dementia) were compared by One-Way ANOVA, and *p*-values were corrected for multiple comparisons using Bonferroni. Data were reported as mean and SD unless stated otherwise.

### Molecular bioinformatics and MBP functional proteomics

MitoMiner was used for analysis of the mitochondria proteome [73]. I-Tasser suite [74] was used for MBP modelling, and Pymol was used to render the 3D structure of the protein [75]. Using previously reported LC-MS/MS data on the byproducts of bovine MBP degradation [43], we performed functional proteomics to investigate the degradation profile of MBP in the temporal lobe of AD + CVD post-mortem brain tissues. Initially, pairwise alignment between bovine and human MBP sequences was performed using EMBOSS Needle [76] (Additional file 7: Figure S2). Semi-trypsin was enabled during database search. Two criteria were applied to detect MBP peptides as degradation byproducts: (1) an “exact match” between the identified peptide and the peptide obtained by pairwise alignment, and (2) the presence of modified Lys/Arg at the terminal sites of the peptides (thereby rejecting the tryptic-digested peptides generated during sample processing). Using these criteria, the MBP degradation byproducts TQDENPVVHF and YLATASTMDHAR# (# referring to Arg citrullination) were identified with ≥99 % confidence. MBP percentage degradation was calculated using the following equation:

$$ \frac{Total\kern0.5em  iTRAQ\kern0.5em  reporter\kern0.5em  area\kern0.5em "\mathrm{exact}\kern0.5em \mathrm{match}"\kern0.5em \times \kern0.5em 100}{Total\kern0.5em  iTRAQ\kern0.5em  reporter\kern0.5em  area\kern0.5em "\mathrm{exact}\kern0.5em \mathrm{match}"\kern0.5em +\kern0.5em  Total\kern0.5em  iTRAQ\kern0.5em  reporter\kern0.5em  area\kern0.5em " partial\kern0.5em  match"} $$

In this equation, “exact match” refers to the identified MBP degradation byproducts described above. “Partial match” refers to longer tryptic peptides that included the exact match sequence of the degradation byproducts. Total iTRAQ reporter area refers to the sum of all identified iTRAQ reporter peak areas for the peptide. The calculation was performed in each group including age-matched controls, women with dementia, and men with dementia. Finally, the degradation ratio was obtained by dividing the MBP percentage degradation values for the women and men dementia groups by that calculated for age-matched controls.

### Western blot

Tissues from two AD + CVD women (68y and 79y respectively), two AD + CVD men (74y and 63y respectively), one male control (77y) and one female control (77y) were homogenized as described above in 1 % SDS buffer. Western blot was performed as previously reported [77]. Briefly, proteins were reduced by 2-mercaptoethanol (5 %) in 95 °C during five minutes and subsequently mixed with BioRad 2× Laemmli sample buffer (CA, USA). Protein amount was quantified by Bradford assay and equal amount of protein was loaded in a 15 % SDS-PAGE gel. Resolved proteins were then blotted onto a nitrocellulose membrane and detected using the polyclonal goat anti-PLP (SC-23570) primary antibody (1:1000 dilution) and the rabbit anti-goat (SC-2768) secondary antibody (1:3000 dilution). Ponceau staining was used as loading control [78]. Densitometry of Western blot signal was mesured using ImageJ (Rasband W.S., ImageJ. U.S. National Institutes of Health, MD., U.S.A. http://imagej.nih.gov/ij/ 1997-2016) and Student's t-test  analysis of data was performed.

### Data deposition

Proteomics data have been made publicly available through ProteomeXchange consortium [79] via the partner repository PRIDE under the following identifier PXD003027.

## Additional files

Additional file 1: Table S1. Tabular data of differential regulated white matter proteome in BA21 of AD + CVD subjects compared to age-matched controls. 1. *p*-value of women/control groups. 2. *p*-value of men/control groups. (DOC 33 kb) Additional file 2: Table S2. Tabular data of differential regulated mitochondrial proteome in BA21 of AD + CVD subjects compared to age-matched controls. 1. *p*-value of women/control groups. 2. *p*-value of men/control groups. (DOCX 16 kb) Additional file 3: Figure S1. A. Percentage coefficient of variation of calculated iTRAQ ratios. Less than 5 % of protein ratios in dementia groups show a %CV higher than 50 % (>1.5 ratio). B. Plot of the post-mortem delay confidence intervals showing no significant differences between the experimental groups which indicates no effect of this variable on the identified proteome changes. C. Number of 99 % of confidence identified peptides included in our study based on the obtained G-test *p*-values after FDR correction (*p* < 0.014). D. Percentage coefficient of variation of G-test significant proteins in AD with CVD women and men groups. 95 % of the proteins showed less than 20 % of differential variation between groups. E. Analysis of age influence on the occurrence of DPTMs (Asn/Gln deamidation and Arg citrullination) in the temporal lobe of AD + CVD versus Control. Younger patient displayed significantly higher levels of DPTMs in whole BA21 proteome compared to a significantly older control. F. Spectral count of MBP DPTMs (Asn/Gln deamidation and Arg citrullination) in the temporal lobe of AD + CVD versus Control. Younger patient displayed significantly higher levels of DPTMs on the MBP profile compared to a significantly older control. (PDF 255 kb) Additional file 4: Dataset containing the total number of temporal lobe proteins that passed the FDR correction applied to the calculated G-test *p*-values. (XLS 140 kb) Additional file 5: Dataset containing the total number of proteins significantly modulated in the temporal lobe of AD+CVD patients compared to controls. (XLS 44 kb) Additional file 6: Dataset containing the total number of proteins significantly modulated by the effect of gender in the temporal lobe of AD+CVD patients. (XLS 37 kb) Additional file 7: Figure S2. Structural alignment of human and bovine MBP sequences. EMBOSS_001 corresponds to human MBP and MBP_Bovine corresponds to bovine MBP. (PDF 231 kb)
