# Supplementary material for: Gender differences in white matter pathology and mitochondrial dysfunction in Alzheimer’s disease with cerebrovascular disease
Source: Mol Brain. 2016 Mar 17;9:27. doi: 10.1186/s13041-016-0205-7 (PMC4794845; doi:10.1186/s13041-016-0205-7)
Supplement: Additional file 1: Table S1. — Tabular data of differential regulated white matter proteome in BA21 of AD + CVD subjects compared to age-matched controls. 1. p-value of women/control groups. 2. p-value of men/control groups. (DOC 33 kb) [file 13041_2016_205_MOESM1_ESM.doc]

| **Name** | **Women/control ratio** | **SD** | **p-value ^1^** | **Men/control ratio** | **SD** | **p-value ^2^** | **Gender difference** |
| --- | --- | --- | --- | --- | --- | --- | --- |
| Neural cell adhesion molecule 1 | 1.31 | 0.16 | 0.220 | 1.56 | 0.39 | 0.035 | 0.24 |
| 2',3'-cyclic-nucleotide 3'-phosphodiesterase | 5.23 | 1.04 | <0.001 | 3.65 | 2.08 | <0.001 | 1.58 |
| Myelin proteolipid protein | 12.81 | 2.60 | 0.002 | 5.87 | 8.58 | 0.014 | 6.94 |
| Myelin basic protein | 1.12 | 1.47 | 0.000 | 1.06 | 1.25 | 0.000 | 0.06 |
| Hyaluronan and proteoglycan link protein 2 | 4.22 | 0.37 | <0.001 | 3.24 | 1.20 | 0.003 | 0.98 |
| Cathepsin D | 0.61 | 0.07 | 0.054 | 0.86 | 0.30 | 0.525 | 0.24 |
|  |  |  |  |  |  |  |  |

**Additional file 6: Table S1.** Tabular data of differential regulated white matter proteome in BA21 of AD+CVD subjects compared to age-matched controls. 1. p-value of women/control groups. 2. p-value of men/control groups.
