# Supplementary material for: Gender differences in white matter pathology and mitochondrial dysfunction in Alzheimer’s disease with cerebrovascular disease
Source: Mol Brain. 2016 Mar 17;9:27. doi: 10.1186/s13041-016-0205-7 (PMC4794845; doi:10.1186/s13041-016-0205-7)
Supplement: Additional file 2: Table S2. — Tabular data of differential regulated mitochondrial proteome in BA21 of AD + CVD subjects compared to age-matched controls. 1. p-value of women/control groups. 2. p-value of men/control groups. (DOCX 16 kb) [file 13041_2016_205_MOESM2_ESM.docx]

**Additional file 7: Table S2.** Tabular data of differential regulated mitochondrial proteome in BA21 of AD+CVD subjects compared to age-matched controls. 1. p-value of women/control groups. 2. p-value of men/control groups.

| **Name** | **Women/control ratio** | **SD** | **p-value ^1^** | **Men/control ratio** | **SD** | **p-value ^2^** | **Gender difference** |
| --- | --- | --- | --- | --- | --- | --- | --- |
| Creatine kinase U-type, mitochondrial | 0.59 | 0.01 | 0.003 | 0.60 | 0.01 | 0.002 | 0.00 |
| Superoxide dismutase [Cu-Zn] | 1.90 | 0.27 | 0.074 | 2.41 | 0.73 | 0.007 | 0.52 |
| Alpha-crystallin B chain | 2.01 | 0.09 | 0.002 | 1.36 | 0.80 | 0.126 | 0.65 |
| Glutamine synthetase | 0.50 | 0.09 | 0.002 | 1.11 | 0.74 | 0.219 | 0.61 |
| Peroxiredoxin-5, mitochondrial | 0.59 | 0.07 | 0.067 | 0.59 | 0.09 | 0.048 | 0.00 |
| Stress-70 protein, mitochondrial | 0.59 | 0.04 | 0.015 | 0.74 | 0.20 | 0.139 | 0.16 |
| ATP synthase subunit d, mitochondrial | 0.60 | 0.04 | 0.010 | 0.96 | 0.45 | 0.567 | 0.36 |
| V-type proton ATPase catalytic subunit A | 1.50 | 0.19 | 0.061 | 2.00 | 0.66 | 0.002 | 0.50 |
| Cytochrome c oxidase subunit 5A, mitochondrial | 0.56 | 0.01 | 0.020 | 0.85 | 0.36 | 0.413 | 0.29 |
| Cytochrome b-c1 complex subunit 7 | 0.53 | 0.01 | 0.016 | 0.88 | 0.43 | 0.516 | 0.35 |
| Aldehyde dehydrogenase, mitochondrial | 0.64 | 0.02 | 0.040 | 0.78 | 0.17 | 0.213 | 0.14 |
| Prohibitin | 0.76 | 0.10 | 0.315 | 0.55 | 0.28 | 0.029 | 0.21 |
| Alpha-aminoadipic semialdehyde dehydrogenase | 1.18 | 0.07 | 0.278 | 1.76 | 0.72 | 0.006 | 0.58 |
| NADH dehydrogenase [ubiquinone] 1 alpha subcomplex subunit 5 | 0.55 | 0.07 | 0.035 | 0.75 | 0.25 | 0.273 | 0.20 |
| ATP synthase subunit O, mitochondrial | 1.71 | 0.10 | 0.027 | 0.97 | 0.90 | 0.691 | 0.74 |
| NADH dehydrogenase [ubiquinone] 1 alpha subcomplex subunit 8 | 0.49 | 0.05 | 0.005 | 0.70 | 0.27 | 0.164 | 0.21 |
| Succinyl-CoA ligase [ADP/GDP-forming] subunit alpha, mitochondrial | 0.78 | 0.03 | 0.278 | 0.55 | 0.28 | 0.018 | 0.22 |
| Pyruvate dehydrogenase E1 component subunit beta, mitochondrial | 0.70 | 0.06 | 0.088 | 0.60 | 0.12 | 0.046 | 0.10 |
