# Supplementary material for: Gender differences in white matter pathology and mitochondrial dysfunction in Alzheimer’s disease with cerebrovascular disease
Source: Mol Brain. 2016 Mar 17;9:27. doi: 10.1186/s13041-016-0205-7 (PMC4794845; doi:10.1186/s13041-016-0205-7)
Supplement: Additional file 3: Figure S1. — A. Percentage coefficient of variation of calculated iTRAQ ratios. Less than 5 % of protein ratios in dementia groups show a %CV higher than 50 % (>1.5 ratio). B. Plot of the post-mortem delay confidence intervals showing no significant differences between the experimental groups which indicates no effect of this variable on the identified proteome changes. C. Number of 99 % of confidence identified peptides included in our study based on the obtained G-test p-values after FDR correction (p < 0.014). D. Percentage coefficient of variation of G-test significant proteins in AD with CVD women and men groups. 95 % of the proteins showed less than 20 % of differential variation between groups. E. Analysis of age influence on the occurrence of DPTMs (Asn/Gln deamidation and Arg citrullination) in the temporal lobe of AD + CVD versus Control. Younger patient displayed significantly higher levels of DPTMs in whole BA21 proteome compared to a significantly older control. F. Spectral count of MBP DPTMs (Asn/Gln deamidation and Arg citrullination) in the temporal lobe of AD + CVD versus Control. Younger patient displayed significantly higher levels of DPTMs on the MBP profile compared to a significantly older control. (PDF 255 kb) [file 13041_2016_205_MOESM3_ESM.pdf]

a

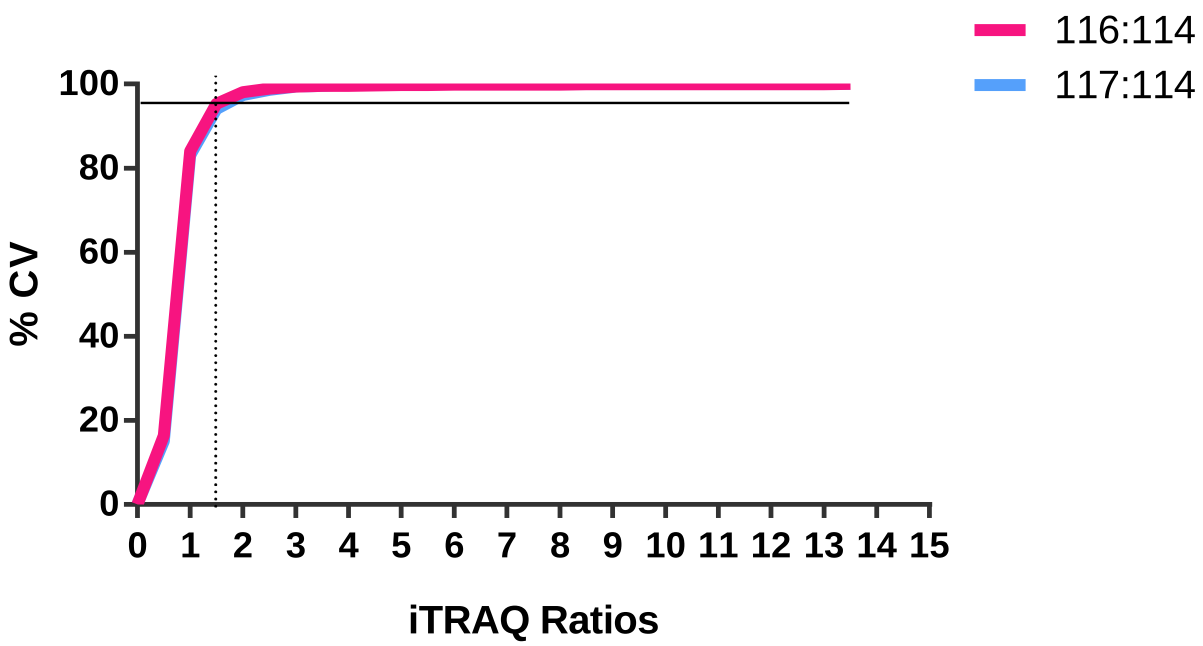
116:114
117:114
100
80
60
40
20
0
% CV
0 1 2 3 4 5 6 7 8 9 10 11 12 13 14 15
iTRAQ Ratios

b

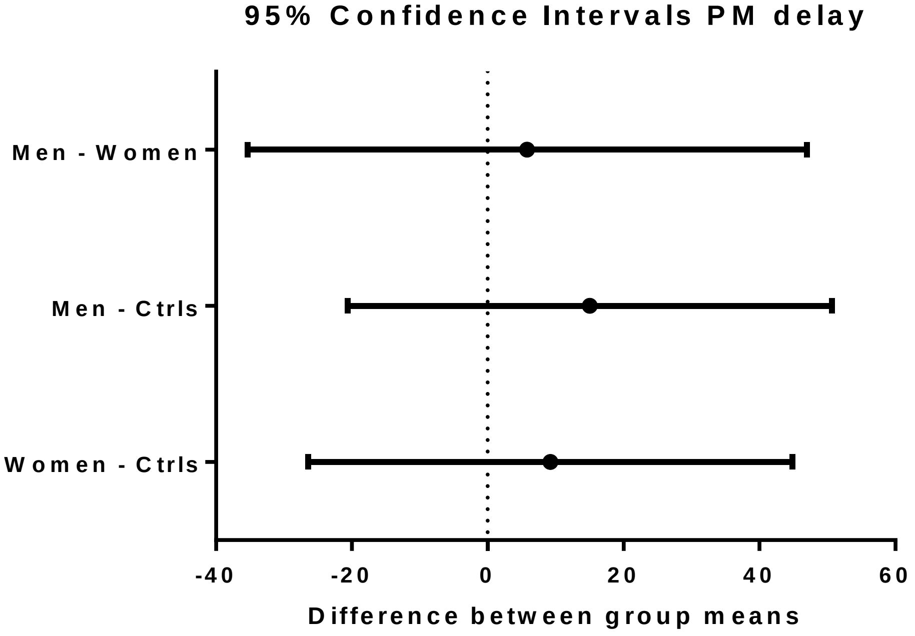
95% Confidence Intervals PM delay
Men - Women
Men - Ctrls
Women - Ctrls
-40 -20 0 20 40 60
Difference between group means

c

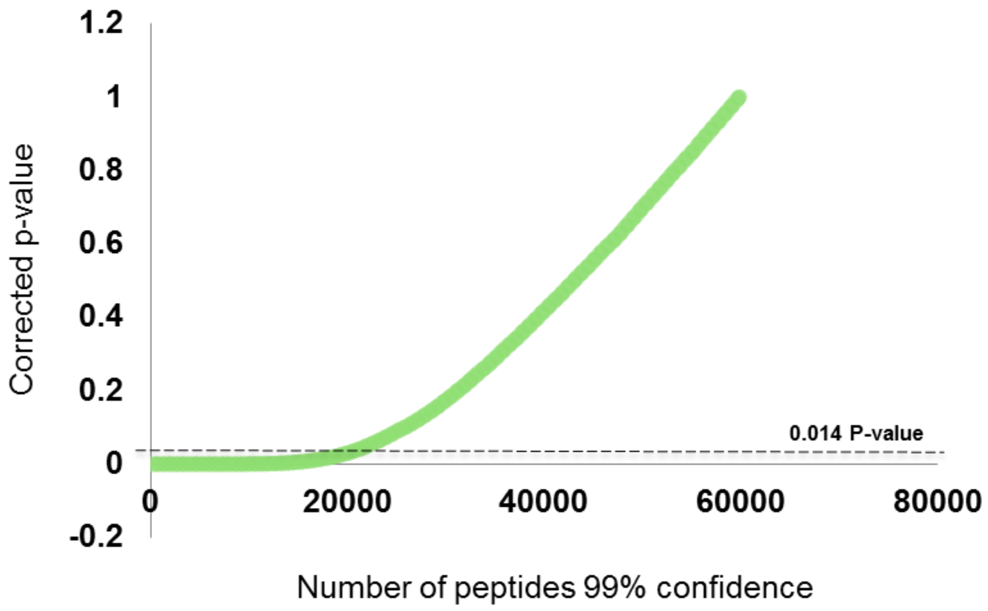

1.2
1
0.8
0.6
0.4
0.2
0
-0.2
Corrected p-value
0.014 P-value
0
20000
40000
60000
80000
Number of peptides 99% confidence

d

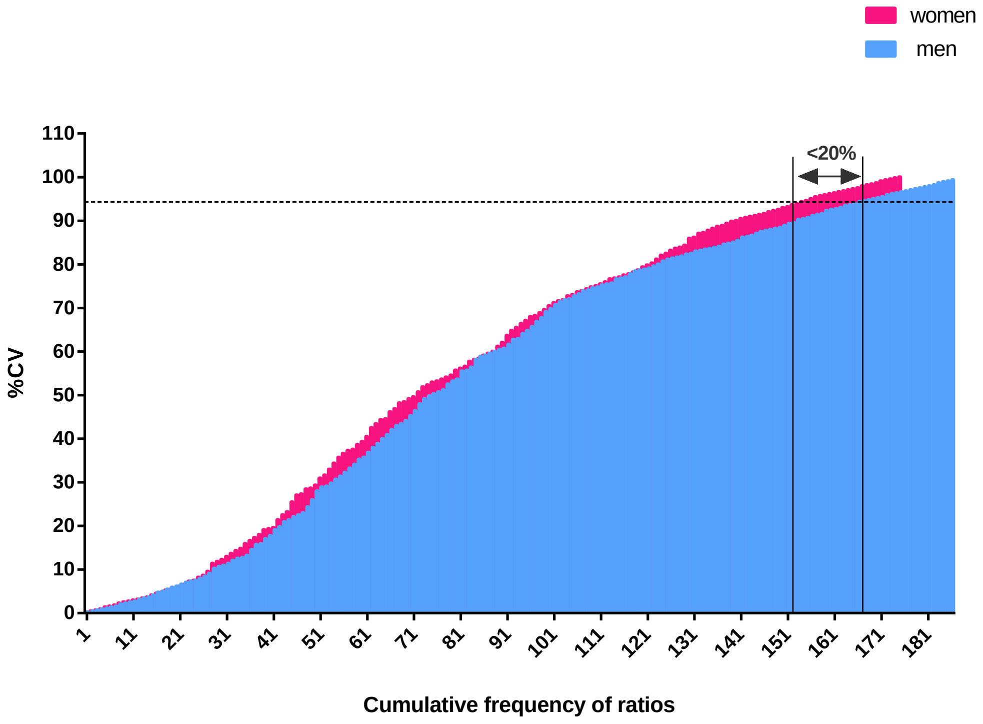

women
men
110
100
90
80
70
60
50
40
30
20
10
0
%CV
<20%
1
11
21
31
41
51
61
71
81
91
101
111
121
131
141
151
161
171
181
Cumulative frequency of ratios

e

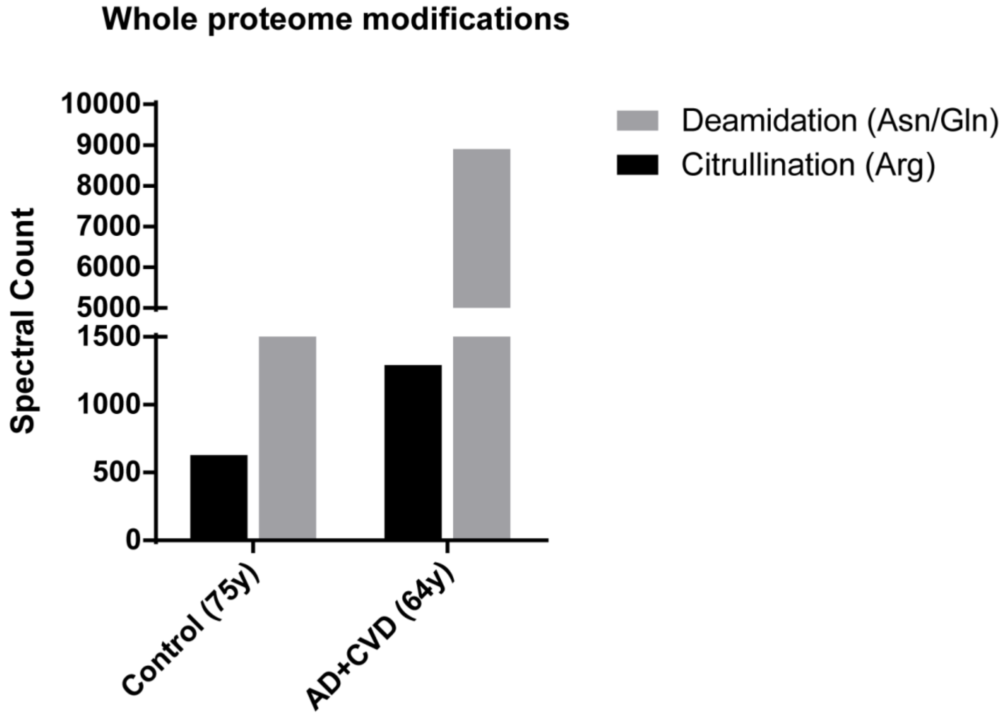

f

Spectral count of MBP modified peptides

PTM	Control	Patient
Deamidation	158	196
Citrullination	202	460

Additional file 1: Figure S1. A. Percentage coefficient of variation of calculated iTRAQ ratios. Less than 5% of protein ratios in dementia groups show a %CV higher than 50% (>1.5 ratio). **B .** Plot of the post-mortem delay confidence intervals showing no significant differences between the experimental groups which indicates no effect of this variable on the identified proteome changes. **C.** Number of 99% of confidence identified peptides included in our study based on the obtained G-test p-values after FDR correction ($p<0.014$). **D.** Percentage coefficient of variation of G-test significant proteins in AD with CVD women and men groups. 95% of the proteins showed less than 20% of differential variation between groups. **E.** Analysis of age influence on the occurrence of DPTMs (Asn/Gln deamidation and Arg

citrullination) in the temporal lobe of AD+CVD versus Control. Younger patient displayed significantly higher levels of DPTMs in whole BA21 proteome compared to a significantly older control. **F.** Spectral count of MBP DPTMs (Asn/Gln deamidation and Arg citrullination) in the temporal lobe of AD+CVD versus Control. Younger patient displayed significantly higher levels of DPTMs on the MBP profile compared to a significantly older control.
