# Supplementary material for: Gender differences in white matter pathology and mitochondrial dysfunction in Alzheimer’s disease with cerebrovascular disease
Source: Mol Brain. 2016 Mar 17;9:27. doi: 10.1186/s13041-016-0205-7 (PMC4794845; doi:10.1186/s13041-016-0205-7)
Supplement: Additional file 7: Figure S2. — Structural alignment of human and bovine MBP sequences. EMBOSS_001 corresponds to human MBP and MBP_Bovine corresponds to bovine MBP. (PDF 231 kb) [file 13041_2016_205_MOESM7_ESM.pdf]

```
MBP_BOVIN          1 --------------------------------------------------      0

EMBOSS_001         1 MGNHAGKRELNAEKASTNSETNRGESEKKRNLGELSRTTSEDNEVFGEAD     50

MBP_BOVIN          1 --------------------------------------------------      0

EMBOSS_001        51 ANQNNGTSSQDTAVTDSKRTADPKNAWQDAHPADPGSRPHLIRLFSRDAP    100

MBP_BOVIN          1 ----------------------------------AAQKRPSQR--SKYLA     14
                                                       |:|||||||  |||||
EMBOSS_001       101 GREDNTFKDRPSESDELQTIQEDSAATSESLDVMASQKRPSQRHGSKYLA    150

MBP_BOVIN         15 SASTMDHARHGFLPRHRDTGILDSLGRFFGSDRGAPKRGSGKDGHHAART     64
                     :|||||||||||||||||||||||:|||||.||||||||||||.||.|||
EMBOSS_001       151 TASTMDHARHGFLPRHRDTGILDSIGRFFGGDRGAPKRGSGKDSHHPART    200

MBP_BOVIN         65 THYGSLPQKAQGHRPQDENPVVHFFKNIVTPRTPPPSQGKGRGLSLSRFS    114
                     .||||||||:.| |.|||||||||||||||||||||||||||||||||||
EMBOSS_001       201 AHYGSLPQKSHG-RTQDENPVVHFFKNIVTPRTPPPSQGKGRGLSLSRFS    249

MBP_BOVIN        115 WGAEGQKPGFGYGGRASDYKSAHKGLKGHDAQGTLSKIFKLGGRDSRSGS    164
                     ||||||:||||||||||||||||||.||.|||||||||||||||||||||
EMBOSS_001       250 WGAEGQRPGFGYGGRASDYKSAHKGFKGVDAQGTLSKIFKLGGRDSRSGS    299

MBP_BOVIN        165 PMARR    169
                     |||||
EMBOSS_001       300 PMARR    304
```

Additional file 5: Figure S2 . Structural alignment of human and bovine MBP sequences. EMBOSS_001 corresponds to human MBP and MBP_Bovine corresponds to bovine MBP.
